# A Direct and Sensitive Method for Determination of 5-Fluorouracil in Colorectal Cancer Cells: Evaluating the Effect of Stromal Cell on Drug Resistance of Cancer Cells

**DOI:** 10.1155/2021/6689488

**Published:** 2021-02-25

**Authors:** Fengjing Xu, Zhipeng Wang, Xinhua Song, Mengwei Zhang, Lili Cui, Yanping Liu, Hongxia Yan, Shouhong Gao, Yan Liu, Wansheng Chen

**Affiliations:** ^1^College of Traditional Chinese Medicine, Yunnan University of Chinese Medicine, Kunming, Yunnan 650500, China; ^2^Department of Pharmacy, Changzheng Hospital, Second Military Medical University, Shanghai 200003, China; ^3^Department of Pharmacy, Xinhua Hospital, Affiliated to Shanghai Jiao Tong University School of Medicine, Shanghai 200092, China; ^4^School of Chemistry and Biology, Yichun College, Yichun City, Jiangxi Province 336000, China

## Abstract

Fibroblasts in the stroma play a critical role in tumor evolution. In this study, we assessed the influence of colonic fibroblasts on colon cancer cells treated with 5-fluorouracil (5-FU), and mouse colon cancer cell lines MC38 and colonic fibroblasts NIH3T3 were used in this study. A sensitive and rapid UHPLC-MS/MS method for the quantitation of 5-FU from the cell and their medium has been successfully developed and validated. The cells were lysed with methanol, and the mixture was evaporated and then redissolved to extract intracellular 5-FU. The analysis was performed on UHPLC-MS/MS using an Atlantis T3-C18 column (3 *μ*m, 2. 1 ∗ 100 mm) and gradient elution with acetonitrile and 0.1% formic acid in water. Method validation included the following parameters: the matrix effect range 88.82%–93.64% and the recovery range 93.52%–94.56%. The intraday and interday precision and accuracy were <11% and within ±6%, and the stability, specificity, carry-over, dilution effect, and linearity all conformed to the criteria. The method was applied to detect the concentration of 5-FU inside cells and cell culture medium. The preliminary results present that NIH3T3 could enhance the drug resistance of MC38 to 5-FU with a decreased intracellular concentration of 5-FU in MC38, which showed a positive relationship with NIH3T3 number.

## 1. Introduction

Colorectal cancer (CRC) is one of the most common cancers worldwide. Its incidence ranks thirdly after prostate cancer and lung cancer among males and is the second most common cancer in females. Chemotherapy intervention combined with surgery is one of the main treatments for advanced CRC. In several decades after its discovery, 5-FU has been the backbone in adjuvant and palliative treatment for colorectal cancer (CRC) [1, 2]. Despite advances in new treatment drugs in recent years, 5-FU is still a first-line agent for CRC patients [3]. However, during the treatment process, most patients will develop drug resistance to 5-FU, which is a major obstacle for CRC treatment [4]. In recent years, drug resistance has stirred up much attention of researchers on the mechanism exploration and resistance elimination.

Nowadays, it has been found that tumor has a complex microenvironment, and tumor progression, metastasis, and drug resistance are largely promoted by the tumor microenvironment, among which stromal cells and extracellular environment are crucial for the occurrence and development of CRC [5–7]. The tumor microenvironment is composed of not only cancer cells but also a variety of stromal cells, for example, cancer-associated fibroblasts (CAFs), bone marrow derived cells, and immune and inflammatory cells. Since Paget proposed the “seed and soil” hypothesis [8], the biological importance of cancer microenvironments has been widely accepted. CAFs, the largest number cell type in tumor stroma, are produced by activating a population of mesenchymal cells in the body, recruiting mesenchymal stem cells and fibroblasts from bone marrow sources. CAFs have multiple functions, including regulation of intestinal inflammation, epithelial cell proliferation, stem cell maintenance, angiogenesis, extracellular matrix remodeling, and metastasis, which in consequence promote the development and progression of CRC [9]. Nowadays, there have many studies on CAF promoted drug resistance, but the variations of 5-FU in stromal cell-mediated colorectal cancer drug resistance have not been clarified [10–12].

The purpose of this study was to establish and validate a direct, sensitive, and effective UHPLC-MS/MS method to quantify 5-FU in medium and cell lysates and evaluate the effects of the different number of stromal cells on 5-FU concentrations in cancer cells.

## 2. Materials and Methods

### 2.1. Chemicals and Materials

5-FU and 5-bromouracil (5-BrU, Internal Standard) were obtained from Meilun Bio-tech Co., Ltd. (Dalian City, China). MS-grade acetonitrile (ACN) and MeOH were obtained from Merck (Merck Company, Darmstadt, Germany). Analytical pure formic acid was purchased from McLean Bio-Tech Co., LTD. (McLean, Shanghai, China). Isopropanol in HPLC-grade was purchased from Shanghai Titan Technology Co., Ltd (Shanghai. China). Distilled water was bought from Shenzhen Watsons Distilled Water Co., Ltd (Shenzhen, China). Vivaspin® 500 ultrafiltration centrifugal concentrator was purchased from Sartorius Trading Co. Ltd. (Sartorius, Shanghai, China). 96-well plates were purchased from Corning Co., Ltd (Corning City, USA). ScanSpeed MaxiVac Alpha/Beta (GVS03511110028) was provided by Gene Company Limited (Danish, Germany).

### 2.2. Cell Culture

MC38 (mouse colorectal cancer cells) was a gift from the Pharmacological Laboratory of Tongji University. NIH3T3 (mouse colorectal fibroblasts) was purchased from Meilun Bio-tech Co., Ltd. (Dalian City, China). These two cell lines were grown in DMEM (Dulbecco's modified Eagle's medium) supplemented with 10% heat-inactivated fetal bovine serum, glutamine, and 1% penicillin-streptomycin at 37°C and 5% CO_2_. The medium was refreshed every 2 or 3 days until reaching an 80%–85% confluence, and the cells were transferred to the next experiment or prepared for stock solution.

### 2.3. Liquid Chromatographic and MS/MS Conditions

The 5-FU quantification was performed on Agilent 1290 series UHPLC system which includes an online degasser, a binary pump, an autosampler, and column oven, and interfaced to an Agilent 6460A triple quadrupole mass spectrometer equipped with an electrospray ionization source (Agilent Technologies, Santa Clara, CA, USA). All raw data are acquired and analyzed using Agilent Masshunter data processing software (version B.06.00, Agilent Technologies, Santa Clara, CA, USA).

### 2.4. Liquid Chromatographic Conditions

Chromatographic separation for 5-FU was achieved using an Atlantis® T3-C18 column (3 *μ*m, 2.1 × 100 mm, Waters Co., Milford, USA), with the temperature maintained at 25°C. Formic acid (0.1%, V/V) in water (A) and ACN (B) was used as mobile phase using the following elution gradient: 0 min, 2% B; 0–3 min, 2% to 70% B; 3.01–3.5 min, 95%-95%B, and a post time of 2 min. Chromatographic separation of analyte was achieved within 3.5 min, with a total run time of 5.5 min. The liquid chromatography system was equilibrated with the initial phase for 10 min before the first injection. The injection volume was 5 *μ*L with a needle wash using 5% MeOH aqueous solution for 3 s.

### 2.5. Mass Spectrometry Conditions

**Figure 1:**
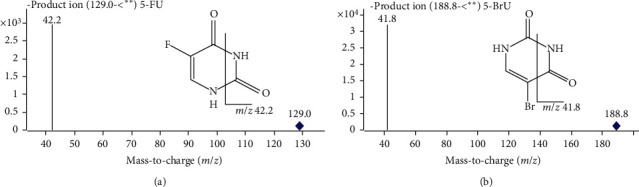
Product ions chromatograms and fragment structures of 5-FU (a) and 5-BrU (b).

The mass detection was optimized based on electrospray ionization source [13] in the positive mode with the capillary voltage set at 4000 V. Drying gas, nebulizer gas, and sheath gas were nitrogen. The drying gas was heated to 325°C and delivered at 10 L/min. The temperature of sheath gas was set at 350°C, and the flow rate was 12 L/min. Nebulizer pressure was optimized at 50 psi. High purity nitrogen that served as collision gas was adjusted at 0.2 MPa. Data acquisition was performed in the selected reaction monitoring (SRM) mode (Figure 1). Mass transitions were monitored at 30 ms dwell time and parameters are listed in Table 1.

### 2.6. Preparation of Standard and Quality Control Samples

Two milligrams of 5-FU was accurately weighed and dissolved in MeOH to obtain the stock solution with a concentration of 1000.0 *μ*g/mL. The solution was further aliquoted and stored in a refrigerator at −80°C until use. The stock solution of 5-FU was diluted with 10% MeOH to obtain a series of working solutions, and 5-FU solutions were diluted 10 times with DMEM to gain the calibration standards at 20, 50, 100, 500, 1000, 2500, 4000, 5000 ng/mL. QC (quality control) samples were prepared separately using the same procedures at concentrations of 50, 1000, and 4000 ng/mL, respectively, and were stored at −80°C before pretreatment. The same method was used to prepare IS stock solution, and 1000.0 *μ*g/mL 5-BrU IS stock solution was finally obtained and stored at −80°C. Before sample pretreatment, 10% MeOH was utilized to dilute the IS solution at a concentration of 1000 ng/mL.

### 2.7. Sample Extraction

The medium samples were mixed thoroughly with 2-fold volume 10% MeOH (containing 1000 ng/mL IS) and then directly ultrafiltered using vivaspin® 500 ultrafiltration tube, and the filtrate was collected for analysis. For 100 *μ*L cell debris sample, a vortex was performed, and then evaporation was completed within 30 min (ScanSpeed MaxiVac Alpha/Beta). The temperature in the evaporation process was set at 4°C with pressure less than 100 pa, and the residual was redissolved with 100 *μ*L MeOH. After vortex for 2 min, the mixture was subjected to a 14500 × g centrifugation for 10 min after adding a 200 *μ*L 10% MeOH (containing 1000 ng/mL IS), and an 80 *μ*L supernatant was transferred to sample vial for analysis.

### 2.8. The Effect of Different Amounts of NIN3T3 Cells on the Drug Resistance of MC38 Treated with the 5-FU

This research protocol was designed to investigate the influence of culture medium (CM) from different amounts of NIH3T3 on the drug resistance of MC38 treated with 5-FU. NIH3T3 was seeded into a 96-well plate for 40 wells and was divided into four groups (10000/mL, 25000/mL, 50000/mL, 75000/mL, and 100 *μ*L/well). All the cells were cultured with full medium overnight. NIH3T3 medium was refreshed with 0.1% BSA (bovine serum albumin) LG DMEM containing 5 *μ*M 5-FU and cultured for another 24 h. The CM of all NIH3T3 cells was harvested and a 1000×g centrifugation was carried out prior to the 5-FU determination and transfer to treat MC38 cells. The NIH3T3 cells were then washed with 1 × PBS two times, and 50 *μ*L/well MeOH was added to all NIH3T3 cells to lyse the cells. The mixtures were all harvested and stored at −80°C until sample pretreatment. MC38 cells were seeded 24 h before the NIH3T3 CM harvest into another 96-well plate for 60 wells and evenly divided into 6 groups, and 4000/mL MC38 cells were seeded per well and cultured with full medium overnight. The medium of MC38 was discarded and 1×PBS was utilized to wash MC38 two times before the NIH3T3 CM was added. The first group of MC38 was the blank control and 0.1% BSA LG DMEM was used to treat the cell. The second group was treated with 0.1% BSA LG DMEM containing 5 *μ*M 5-FU. The other four groups were treated with the CM from different groups of NIH3T3, respectively. All the medium and MC38 cells were collected after 24 h using the method mentioned above.

### 2.9. Method Development

In this study, according to FDA and Chinese Pharmacopoeia (2015 edition), the specificity, linearity, daytime precision and accuracy, and residual effect were verified. We adopted the method of Wang et al., 2018 [14].

On the specificity side, we compared the responses of at least 6 different batches of pointed doses with blank samples. Six replicates were prepared, at low and high concentrations (50 and 4000 ng/mL, low and high), to assess matrix effects and recovery. Five replicates were prepared at 4 concentration levels (low limit, low, medium, high), and the precision and accuracy of the day and day samples were evaluated in 3 batches at different times. The linear evaluation shall be performed three times, and for each concentration point, the inverse calculated concentration deviation of the corresponding calibration standard shall be within 15% (RE%) and the lower limit (RE%) shall be within 20%. The residual effect of the analyte was tested by injecting the highest calibration standard sample. The comparison between the actual measured concentration and the measured concentration serves as a dilution effect. Each dilution factor should be evaluated at least 5 times, with RSD% and RE% not exceeding 15% and 15%, respectively. Stability tests include short-term stability (24 hours in the automatic sampler), long-term stability (1 month), and freeze-thaw cycle stability. QC samples are evaluated at low and high concentrations, not exceeding 15% (RE%) from the nominal concentration.

### 2.10. Data Analysis

The statistical analysis and plotting were carried out using SPSS software (IBM Corporation, NY, USA, version 19.0.0) and GraphPad Prism (GraphPad Software, Inc., Chicago, CA, USA, version 7.01). One-way ANOVA and LSD test were performed for comparisons among groups. *P* value <0.05 was considered to have statistical significance.

## 3. Results and Discussion

### 3.1. Chromatographic and Mass Spectrometric Conditions Optimization

To find better parameters that suit our analyte, a series of tests were performed. The detections of 5-FU and IS by mass spectrometry were optimized under electrospray ionization (ESI) mode, which showed superiority in ionization efficiency compared to atmospheric pressure chemical ionization (APCI) sources [15]. As the 5-FU is a polar compound with high hydrophilicity (Log *P* −0.786), and a column with a stronger retention characteristic is required. After testing different columns, for instance, ZORBAX SB-C18, Waters Atlantis T3-C18, Xselect BEH, Xbridge BEH, and Eclipse PLUS-C18, the results found that the 5-FU has better peak shape and retention time in the Waters T3-C18 column, and the other columns did not show satisfying retention and symmetrical peaks. Furthermore, a lower column temperature (25°C) was beneficial to the 5-FU retention compared to a higher temperature (35°C). The retention time of 5-FU was optimized to 2 min after the 98% water phase was applied in the initial mobile phase. Different mobile phase additives such as ammonium acetate, formic acid, and acetic acid were tested in the process of chromatographic conditions development, and finally, a 0.1% formic acid (V : V) in water and ACN presented a better response of 5-FU. The ammonium acetate could suppress signal response, and MeOH decreased the symmetry of peaks. Mass spectrometry parameters including drying gas, sheath gas, and capillary voltage were optimized in sequence to obtain a better response of 5-FU. The results are listed in Section 2.4 and Table 2.

### 3.2. Sample Extraction

For intracellular drug extraction, the cells were lysed by the extraction solvent (MeOH), and the cell debris was collected together with 5-FU. A few more steps were carried out to further remove the interferents because simple centrifugation and/or filtration could not complete this elimination. Before the evaporation, a standard solid-phase extraction (SPE) with Osis HLB, MCX, or MAX cartridge (Waters Co., Milford, USA) was tested, but low recovery (all <50%) was gained in SPE. And the simple protein precipitation was tested using MeOH (1 : 3, V : V), ACN (1 : 2, V : V), acetone (1 : 2, V : V), or 10% trifluoroacetic acid (1 : 1, V : V), but the response of 5-FU was suppressed severely by the cell debris. Finally, the sample was evaporated first to remove the volatile compounds and the residual was reconstituted with MeOH and IS solution and then subjected to centrifugation to obtain a satisfying matrix effect and recovery.

### 3.3. Method Validation

#### 3.3.1. Specificity

**Figure 2:**
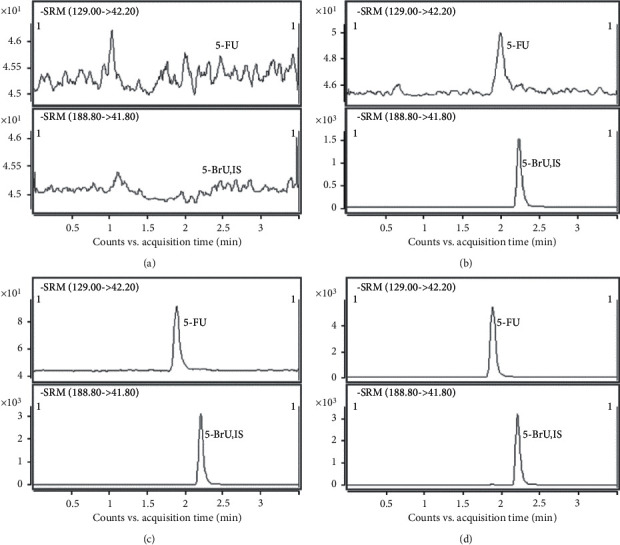
Representative SRM chromatograms for the specificity of 5-FU. (a) Blank sample; (b) blank sample spiked with IS; (c) LLOQ sample; (d) real sample.

The retention time for 5-FU and IS was 1.92 and 2.23 min, respectively. After comparing the responses from blank, IS spiked, LLOQ, and real samples (Figure 2), there were no significant disturbances as the responses in the blank sample <20% of the 5-FU in the LLOQ sample and 5% of IS.

#### 3.3.2. Linearity of Calibration Curves and LLOQ

Calibration curves were obtained by calculating the peak area ratio (analyte/IS) versus nominal concentrations. Eight calibration standards were obtained from the spiked samples, and the best linear and least square residuals were gained under the weighting factor of 1/*χ*^2^. The linear correlation coefficient was over 0.99 for 5-FU. The typical regression equation is as follows: *Y* = 0.001163 ∗ *x* + 0.006233, *r* = 0.9976, LLOQ = 20 ng/mL. The LLOQ is the lowest point of the calibration curve. Linear range 20–5000 ng/mL, weight factor 1/*χ*^2^. Back-calculation deviations are all within ±15%.

#### 3.3.3. Carry-Over and Dilution Effect

**Figure 3:**
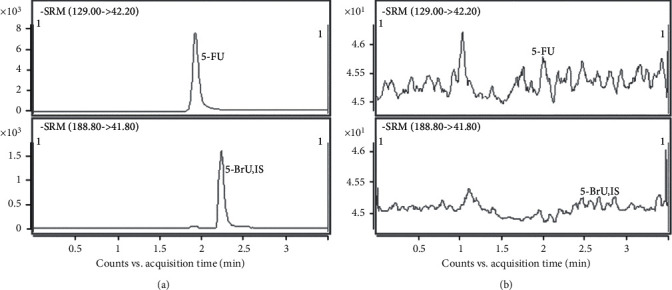
Comparative chromatograms of carry-over of 5-FU. (a) Highest calibration standard; (b) blank sample.

Carry-over between samples often results in confusion in the latter one, especially when the latter one has a much lower concentration. In this method, the highest calibration standards and blank samples were injected orderly for three cycles to evaluate the responses of the analyte in the blank sample. The results show that there is not an obvious response in the blank sample (Figure 3). To assess the dilution effect, five replicate spiked samples of 10000 ng/mL were freshly prepared and diluted with a medium by 5 times and measured by this method, and the RE% and RSD% of dilution factor was within ±15% and less than 15%.

#### 3.3.4. Interday and Intraday Precision and Accuracy

The LLOQ, low, middle, and high spiked samples (20, 50, 500, and 4000 ng/mL) were selected and to analyze the inter- and intraday precision and accuracy. The intraday precision of 5-FU was 1.03–3.96%, intraday accuracy −0.77–3.60%, intraday precision 2.24–5.5%, and intraday accuracy −0.79–3.74%, all of which met the requirements of pharmacopoeia (Table 3).

#### 3.3.5. Matrix Effect and Recovery

The matrix effect and extraction recovery of pretreatment were investigated in low and high QC samples (50 and 4000 ng/mL) in six replicates (using the medium as a matrix). The matrix effect of 5-FU ranged from 88.82% to 93.64%, and the extraction recovery of 5-FU ranged from 93.52% to 94.56%. The RSD% of the IS-adjusted matrix factor ranges from 1.01%–7.92% and for extraction recovery, it ranges from 0.81%–3.68%. Results of matrix effect and extraction recovery are shown in Table 4.

#### 3.3.6. Stability

The results of the stability studies were summarized in Table 5, which includes the long-term stability (one month at -80°C), short-term stability (24 h in autosampler), and three cycles of frozen-thaw stability. The deviations of measured concentration compared to the nominal concentration of the analytes are within ±15%.

### 3.4. The Effect of CM from Different Amounts of NIH3T3 Cell Drug Resistance of MC38

**Figure 4:**
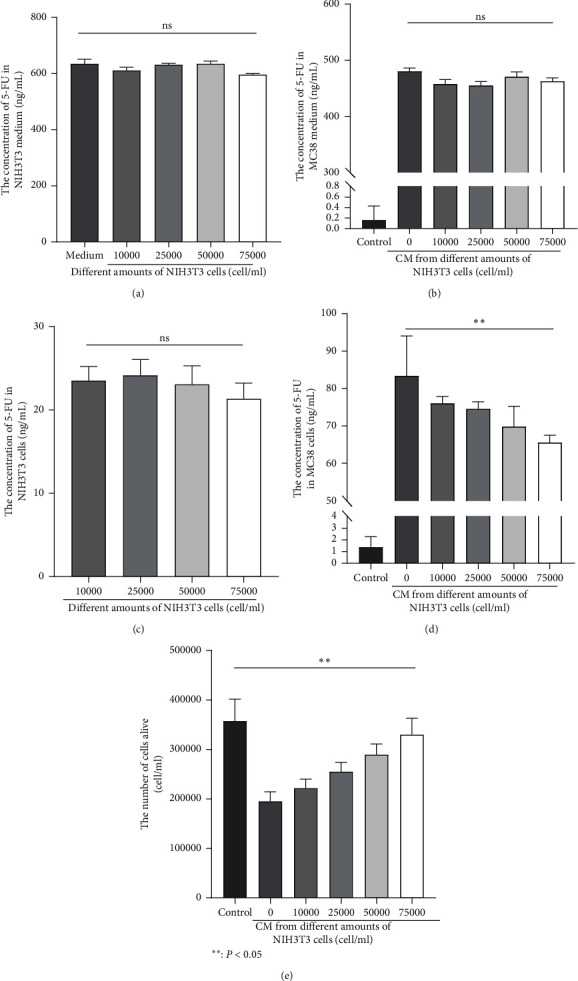
The concentration of 5-FU was measured in medium and in cells. (a) The concentration of 5-FU in the medium of 3T3 cells treated with 5-FU. (b) The concentrations of 5-FU in MC38 medium after a 24-hour treating with NIH3T3 CM. (c) The concentrations of 5-FU in NIH3T3 were determined after treatment with 5-FU. (d) The concentrations of 5-FU in MC38was determined 24 h after the treatment with NIH3T3 CM. (e) NIH3T3 increased the resistance of MC38 to 5-FU.

To explore the amounts of NIH3T3 on MC38 drug resistance, we treated different numbers of stromal cells with 5-FU at 5 *μ*M for 24 h, and the CM was collected and transferred to treat MC38 cells. The concentration of 5-FU was determined in MC38 and NIH3T3 cells and in their medium, the number of these two cells was quantified with MTT (3-(4, 5-dimethylthiazol-2-yl)-2, 5-diphenyltetrazolium bromide) tetrazolium assay. We previously utilized different concentrations of 5-FU solution to treat MC38, and the IC50 value was calculated as 5 *μ*M (data not shown). As shown in Figure 4, the concentration of 5-FU in NIH3T3 medium was not significantly changed among different groups, which proved that the number of NIH3T3 had no effect on the 5-FU concentration in its medium (Figure 4(a)) and intracellular (Figure 4(c)), and in MC38 medium, no differences were found among different groups (Figure 4(b)), but a significantly lower concentration of 5-FU was found inside MC38 cells when treated with CM from more NIH3T3 cells (*P* < 0.01) (Figure 4(d)), and more number of MC38 was quantified in groups treated with CM from more NIH3T3 cells (*P* < 0.05) (Figure 4(e)). Our results prove that NIH3T3 could enhance the drug resistance of MC38 to 5-FU, which had a positive correlation with the NIH3T3 number. The efflux of 5-FU in MC38 cells improved by NIH3T3 might be the potential mechanism.

Recent years have witnessed many studies that focus on the 5-FU and colon cancer cells [16–20]. The drug resistance against 5-FU chemotherapy [21–24] has become a serious problem in clinical practice, and nearly half of metastasis CRC patients developed drug resistance to 5-FU, and the underlying mechanisms include (1) the gene mutations of metabolic enzymes; (2) the enhancement of drug efflux; (3) the improvement of stemness of cancer cells; (4) stromal cell-mediated drug resistance of cancer cells. Lotti et al. [25] prove that the stromal cells treated with chemotherapeutic drugs (FOLFOX) could secret IL-17A to promote the cancer-initiating cells' self-renewal and invasion [26]. Hitomi et al. [27] found that tumor associated macrophages induce resistance to 5-FU chemotherapy in CRC through the release of putrescine. Wang WM et al. reported that fibroblasts secrete cystine and glutathione to help the cancer cell efflux platinum, which could be abrogated by effector T cells. In our preliminary study, we tracked the variations of 5-FU between NIH3T3 and MC38 cells and found that NIH3T3 may enhance the 5-FU efflux of MC38. A further study is necessary to elucidate the specific mechanism that NIH 3T3 mediate the drug resistance of MC38 to 5-FU, and the results may pave the way to the optimization of 5-FU-based chemotherapy in clinical practice.

## 4. Conclusion

In this study, a sensitive and rapid UHPLC-MS/MS method for the determination of 5-FU in cells and medium was established and validated. The calibration curve ranged from 20 to 5000 ng/mL, fully covering the drug concentration in colon cancer cell lines. This method has successfully been applied in determining the intracellular and extracellular 5-FU concentrations in MC38 cells treated by CM from different numbers of NIH3T3 cells. The NIH3T3 could enhance the drug resistance of MC38 to 5-FU, which showed a positive relationship with NIH3T3 number, and further study is needed to uncover the mechanism that stromal cell mediates the drug resistance of cancer cells to 5-FU.

## Figures and Tables

**Table 1:** Mass spectrometric parameters of analyte and internal standard (IS).

Analytes	SRM transition m/*z* (*Q*1 ⟶ *Q*3)	Fragmentor (*V*)	CE (eV)	Retention time (min)
5-FU	129.0 ⟶ 42.2	90	17	1.919
5-BrU	188.8 ⟶ 41.8	90	21	2.228

**Table 2:** The regression parameters of calibration curves of analytes.

Analyte	Regression type	Linear range	Weighing factor	Regression equations	*R* ^2^
5-FU	Linearity	20–5000 ng/mL	1/*χ*^2^	*Y* = 0.001163 ∗ *x* + 0.006233	0.9976

*R*: the correlation coefficient.

**Table 3:** Interday and intraday precision and accuracy of 5-FU (*n* = 5).

Analyte	Nominal concentration (ng/mL)	Interday measured concentration (ng/mL) ±SD	RSD (%)	RE (%)	Intraday measured concentration (ng/mL) ±SD	RSD (%)	RE (%)
5-FU	20	18.41 ± 0.64	3.48	3.60	18.96 ± 1.04	5.50	3.74
	50	52.90 ± 2.10	3.96	1.12	53.34 ± 1.68	3.15	1.34
	500	524.94 ± 5.39	1.03	−0.77	518.36 ± 11.61	2.24	−0.79
	4000	3791.21 ± 45.09	1.19	−0.96	4059.59 ± 102.83	2.47	−0.94

RE: relative error, which is calculated as (measured concentration − nominal concentration)/nominal concentration.

**Table 4:** Extraction recovery and matrix effect of 5-FU.

Analyte	Nominal analyte concentration (ng/mL)	Recovery		Matrix effects	
		Mean (%)±SD	RSD (%)	Mean (%)±SD	RSD (%)
5-FU	50	93.52 ± 0.03	3.68	88.82 ± 0.06	7.29
	4000	94.56 ± 0.01	0.81	93.64 ± 0.01	1.01

**Table 5:** The stability of analytes in different conditions (*n* = 5).

Analyte	Nominal analyte concentration (ng/mL)	Freeze-thaw stability		Short-term stability		Long-term stability	
		Measured concentration (ng/mL)	RE%	Measured concentration (ng/mL)	RE%	Measured concentration (ng/mL)	RE%
5-FU	50	41.32	82	47.50	95	44.17	88
	4000	3872.49	96	3942.21	98	3325.85	83

## Data Availability

The data used to support the findings of this study are available from the corresponding author upon request.

## Abbreviations

5-FU: 5-Fluorouracil
UHPLC-MS/MS: Ultrahigh-performance liquid chromatography tandem mass spectrometry
CRC: Colorectal cancer
CAF: Cancer-associated fibroblasts
MC38: Mouse colorectal cancer cells
NIH3T3: Mouse colorectal fibroblasts
5-BrU: 5-Bromouracil
IS: Internal standard
ACN: Acetonitrile
DMEM: Dulbecco's modified Eagle's medium
SRM: Selected reaction monitoring
BSA: Bovine serum albumin
CM: Culture medium
MTT: 3-(4, 5-Dimethylthiazol-2-yl)-2, 5-diphenyltetrazolium bromide
LLOQ: Lower limit of quantification
ESI: Electrospray ionization
APCI: Atmospheric pressure chemical ionization.

## References

[1] Thanikachalam K., Khan G. (2019). Colorectal cancer and nutrition. *Nutrients*.

[2] McQuade R. M., Stojanovska V., Bornstein J. C., Nurgali K. (2017). Colorectal cancer chemotherapy: the evolution of treatment and new approaches. *Current Medicinal Chemistry*.

[3] Yan L., Yu H.-H., Liu Y.-S., Wang Y.-S., Zhao W.-H. (2019). Esculetin enhances the inhibitory effect of 5-Fluorouracil on the proliferation, migration and epithelial-mesenchymal transition of colorectal cancer. *Cancer Biomarkers*.

[4] Amirkhah R., Farazmand A., Irfan-Maqsood M., Wolkenhauer O., Schmitz U. (2015). The role of microRNAs in the resistance to colorectal cancer treatments. *Cellular and Molecular Biology (Noisy-Le-Grand)*.

[5] Meurette O., Mehlen P. (2018). Notch signaling in the tumor microenvironment. *Cancer Cell*.

[6] Maykel J., Liu J. H., Li H., Shultz L. D., Greiner D. L., Houghton J. (2014). NOD-scidIl2rg tm1Wjl and NOD-rag1 null Il2rg tm1Wjl:a model for stromal cell-tumor cell interaction for human colon cancer. *Digestive Diseases and Sciences*.

[7] Hanahan D., Weinberg R. A. (2011). Hallmarks of cancer: the next generation. *Cell*.

[8] Ishii G., Ochiai A., Neri S. (2016). Phenotypic and functional heterogeneity of cancer-associated fibroblast within the tumor microenvironment. *Advanced Drug Delivery Reviews*.

[9] Koliaraki V., Pallangyo C. K., Greten F. R., Kollias G. (2017). Mesenchymal cells in colon cancer. *Gastroenterology*.

[10] He J., Xie G., Tong J. (2014). Overexpression of microRNA-122 re-sensitizes 5-FU-resistant colon cancer cells to 5-FU through the inhibition of PKM2 in vitro and in vivo. *Cell Biochemistry and Biophysics*.

[11] Peng K., Dinges L.-A., Helm O. (2018). Nuclear factor E2-related factor-2 has a differential impact on MCT1 and MCT4 lactate carrier expression in colonic epithelial cells: a condition favoring metabolic symbiosis between colorectal cancer and stromal cells. *Oncogene*.

[12] Ammar L., De Sousa E Melo F., van der Heijden M. (2010). Wnt activity defines colon cancer stem cells and is regulated by the microenvironment. *Nature Cell Biology*.

[13] Cameron Z., Li X., Yang Y. (2019). A sensitive and efficient method for determination of capecitabine and its five metabolites in human plasma based on one-step liquid-liquid extraction. *Journal of Analytical Methods in Chemistry*.

[14] Wang Z., Yang Y., Zhang F. (2018). A direct, sensitive and efficient method for determination of alpha-fluoro-beta-alanine in urine: evaluating the influence of magnesium isoglycyrrhizinate on excretion in rat model. *Journal of Chromatography B*.

[15] Rebane R., Kruve A., Liigand J., Gornischeff A., Leito I. (2019). Ionization efficiency ladders as tools for choosing ionization mode and solvent in liquid chromatography/mass spectrometry. *Rapid Communications in Mass Spectrometry*.

[16] Liigand L., Zhang S., Li H. (2018). The influence of gut microbiota dysbiosis to the efficacy of 5-Fluorouracil treatment on colorectal cancer. *Biomedicine & Pharmacotherapy*.

[17] Yang H., Kim S. Y., Lee E. (2019). Sex-dependent adverse drug reactions to 5-fluorouracil in colorectal cancer. *Biological and Pharmaceutical Bulletin*.

[18] Lee B., Çapci A., Klein V. (2019). Combination of 5-fluorouracil and thymoquinone targets stem cell gene signature in colorectal cancer cells. *Cell Death Disease*.

[19] Huang R., Lin J. Y., Chi Y. J. (2018). MiR-519d reduces the 5-fluorouracil resistance in colorectal cancer cells by down-regulating the expression of CCND1. *European Review for Medical and Pharmacological Sciences*.

[20] Zhang Q., Li W., Liu G., Tang W. (2019). MicroRNA-24 regulates the growth and chemosensitivity of the human colorectal cancer cells by targeting RNA-binding protein DND1. *J BUON*.

[21] Miyoshi S., Tsugawa H., Matsuzaki J. (2018). Inhibiting xCT improves 5-fluorouracil resistance of gastric cancer induced by CD44 variant 9 expression. *Anticancer Research*.

[22] Hirata J., Wang Y., Lei J., Lei W., Xiong J. P. (2017). Insights into the involvement of noncoding RNAs in 5-fluorouracil drug resistance. *Tumour Biology*.

[23] Conteduca V., Gurioli G., Rossi L. (2018). Oxaliplatin plus leucovorin and 5-fluorouracil (FOLFOX-4) as a salvage chemotherapy in heavily-pretreated platinum-resistant ovarian cancer. *BMC Cancer*.

[24] Riahi-Chebbi I., Souid S., Othman H. (2019). The Phenolic compound Kaempferol overcomes 5-fluorouracil resistance in human resistant LS174 colon cancer cells. *Sci Rep*.

[25] Jeught K. V. d., Xu H.-C., Li Y.-J., Lu X.-B., Ji G. (2018). Drug resistance and new therapies in colorectal cancer. *World Journal of Gastroenterology*.

[26] Lotti F., Jarrar A. M., Pai R. K. (2013). Chemotherapy activates cancer-associated fibroblasts to maintain colorectal cancer-initiating cells by IL-17A. *Journal of Experimental Medicine*.

[27] Hitomi X., Chen Y., Hao L. (2016). Macrophages induce resistance to 5-fluorouracil chemotherapy in colorectal cancer through the release of putrescine. *Cancer Letters*.

